# The role of peer victimization in the physical activity and screen time of adolescents: a cross-sectional study

**DOI:** 10.1186/s12887-017-0913-x

**Published:** 2017-07-19

**Authors:** Jodie A. Stearns, Valerie Carson, John C. Spence, Guy Faulkner, Scott T. Leatherdale

**Affiliations:** 1 Faculty of Physical Education and Recreation, University of Alberta, 1-113 Van Vliet Complex, Edmonton, AB T6G 2H9 Canada; 2 School of Kinesiology, Faculty of Education, University of British Columbia, Vancouver, Canada; 3 School of Public Health and Health Systems, University of Waterloo, Waterloo, Canada

**Keywords:** Negative peer experiences, Peer victimization, Mediation, Adolescents, Youth, Adolescents, Physical activity, Screen time, Sedentary behavior

## Abstract

**Background:**

Negative peer experiences may lead adolescents with overweight and obesity to be less active and engage in more sitting-related behaviors. Our study is among the first to empirically test these associations and hypothesized that 1) peer victimization would mediate the negative association between body weight status and moderate-to-vigorous physical activity (MVPA), and 2) peer victimization would mediate the positive association between body weight status and screen time. Differences by gender were also explored.

**Methods:**

Participants were a part of the Year 1 data (2012–2013) from the COMPASS study, a prospective cohort study of high school students in Ontario and Alberta, Canada. The final sample consisted of 18,147 students in grades 9 to 12 from 43 Ontario secondary schools. The predictor variable was weight status (non-overweight vs. overweight/obese), the mediator was peer victimization, and the outcome variables were screen time and MVPA. Multilevel path analysis was conducted, controlling for clustering within schools and covariates. A few differences were observed between males and females; therefore, the results are stratified by gender.

**Results:**

For both males and females peer victimization partially mediated the association between weight status and screen time. Specifically, females with overweight/obesity reported 34 more minutes/day of screen time than did females who were not overweight and 2 of these minutes could be attributed to experiencing peer victimization. Similarly, males who were overweight/obese reported 13 more minutes/day of screen time than the males who were not overweight and 1 of these minutes could be attributed to experiencing more victimization. Males and females who were overweight/obese also reported less MVPA compared to those who were not overweight; however, peer victimization did not mediate these associations in the hypothesized direction.

**Conclusions:**

We found that higher rates of peer victimization experienced by adolescents with overweight and obesity partially explained why they engaged in more screen time than adolescents who were not overweight. However, the effects were small and may be of limited practical significance. Because this is one of the first studies to investigate these associations, more research is needed before bully prevention or conflict resolution training are explored as intervention strategies.

## Background

Participating in regular physical activity (PA) is important for maintaining a healthy body weight, overall cardiovascular and psychological health, and motor skill development in children and adolescents [1, 2]. Limiting time spend sitting (i.e., sedentary behavior) is also important for the health of young people [3]. Screen-related behaviors in particular, which are often done while sitting, are known to be associated with poor health outcomes. For instance, a recent review found that overall screen time and/or different screen-related behaviors (e.g., TV viewing, playing video games) were associated with unhealthy body composition, cardiometabolic risk, and behavioral conduct, and lower levels of fitness, pro-social behavior, and self-esteem [4]. Despite the known benefits of healthy active living, 95% of Canadian adolescents (aged 12–17 years) are insufficiently active and 76% engage in excessive screen time [5]. Adolescents who are overweight or obese may be particularly vulnerable as they tend to exhibit even lower rates of PA and higher rates of screen time compared to their non-overweight counterparts [6, 7]. This is a particular concern because those who establish unhealthy habits early on in life tend to maintain them into adulthood [8–10]. To inform interventions and health promotion programs, it is important to gain an understanding of why adolescents who are overweight or obese tend to be less active and engage in higher levels of screen time.

Extensive research demonstrates how low PA and excessive screen time are risk factors for overweight/obesity [1, 4]. However, youth who are overweight or obese also face unique barriers, including weight stigma and discrimination that increases their vulnerability to unhealthy behaviors, and perpetuates a “vicious cycle” for these individuals [11–13]. Salvy and colleagues [11] recently proposed a theoretical framework describing the association between overweight/obesity (i.e., body weight status) and PA, and sedentary behavior, and the negative role that peers can play on these associations in young people. Specifically, peer social context, including the presence or absence of peer adversity (e.g., peer victimization, peer rejection) and social isolation (e.g., ostracism, loneliness), is proposed to mediate the negative association between body weight status and PA and the positive association between body weight status and sedentary behavior. Testing this model could provide important insights into interventions designed to get youth who are overweight and obese moving more and away from screens, such as school-level bully prevention programs or conflict resolution training.

Bullying is one aspect of the peer social context that is of particular concern. It is described as an “aggressive goal directed behavior that harms another individual within the context of a power imbalance” [14]. Forms of bullying include verbal (e.g., teasing), physical (e.g., hitting) and relational attacks (e.g., spreading rumors). Bullying can occur in person or through the internet or other computer technology (e.g., texting, emails, social network sites); the latter of which is described as “cyberbulling” [15]. The experience of being bullied is called “peer victimization” and is the focus of this study.

Research has shown that youth that are overweight or obese are more likely to experience peer victimization [16–21]. Specifically, their excess body weight is a physical characteristic that makes them stand out from their peers, putting them at increased risk for being victimized [22]. For example, in a large sample of Canadian adolescents aged 11 to 16 years old, Janssen et al. [18] observed rates of peer victimization to be 10.7%, 14.4%, and 18.5% in healthy weight, overweight, and obese participants, respectively. Further, a recent meta-analysis confirmed this association does not differ by gender [21]. Adolescents perceive that weight-related stigma is the primary reason that peer victimization occurs, and verbal attacks are the most common type of victimization (e.g., made fun of, called names, teased) [23]. Among a sample of adolescents seeking weight-loss treatment, 64% had experienced weight-based victimization and, of these participants, 78% had endured the teasing/bulling for one year, and 36% had experienced the attacks for five years, with peers (92%) and friends (70%) being the most common perpetrators [24].

Bullying often occurs in PA settings. For instance, Puhl and others [23] found that 85% of participants in their study had witnessed weight-based teasing during PA and 58% had observed this behavior at least sometimes, often, or very often. Observational studies reveal that higher rates of peer victimization is associated with lower physical education (PE) attendance, and less PA [25–27], and weight criticism during sports and PA is associated with lower sport enjoyment and lower participation in mild-intensity PA [28]. Further, a recent systematic review of 15 qualitative studies found that adolescents with overweight or obesity reported peer victimization, including social exclusion, stereotyping, verbal bullying, and physical bullying, as barriers to PA participation [13].

Two studies from the Youth Risk Behavior Survey suggest that negative peer experiences can lead to higher levels of screen time in adolescents in grades 9–12 [29, 30]. One found that being bullied in the last 12 months was associated with reporting ≥3 h of TV viewing per day in males, and ≥3 h per day of computer use in both males and females in grades 9–12 [29]. The other observed that females who were bullied on school property in the last 12 months had an increased odds of accumulating ≥3 h/day of video game/computer use, although no associations were found for males. Thus, it seems plausible that higher levels of peer victimization experienced by adolescents with overweight and obesity may help explain why they tend to shy away from activity and stray towards screen-based behaviors.

There are several potential reasons why greater peer victimization may lead to less PA and greater time in sitting-related behaviors. Salvy and authors [11] proposed that negative peer interactions elicit psychological “pain” which impairs executive function and induces apathy. As the individual tries to cope with the pain, they may be more likely to choose sedentary activities such as screen-based behaviors. Those who experience peer victimization may also avoid PA settings due to fear of being bullied, reduced enjoyment of PA, and/or because they are socially excluded and/or not invited to participate in PA activities [13, 27, 28].

To our knowledge, the framework proposed by Salvy et al. (2012) has yet to be empirically tested. Though the causal pathways cannot be rigorously tested in cross-sectional designs [31], such studies can be useful as a first step in obtaining a snapshot of concurrent associations and to justify the need for conducting longitudinal studies [32]. The first aim of the study was to examine whether peer victimization mediates the negative association between body weight status and PA in adolescents. The second aim was to investigate whether peer victimization mediates the positive association between body weight status and screen time in adolescents. Consistent with the theoretical framework by Salvy et al. [11], it was hypothesized that peer victimization would mediate the associations between body weight status and both PA and screen time. Because some differences exist between males and females in the literature, differences by gender were also explored.

## Methods

### Design and procedure

This cross-sectional study uses data from Year 1 (2012–2013 school year) of the COMPASS study. COMPASS is a prospective cohort study designed to annually collect hierarchical longitudinal data from a convenience sample of 24,173 grade 9 to 12 students attending 43 secondary schools in Ontario, Canada. Eligible students were recruited via an active-information passive-consent procedure. Parents were mailed an information letter, and were told to contact the COMPASS research coordinator if they did not want their child to participate. This procedure allowed us to obtain robust data, achieve higher participation rates (82.1% participation rate among eligible students), and maintain student confidentiality. Eligible students willing to participate provided their assent and completed surveys during class time. Eligible students could withdraw or decline to participate at any time, and were assured that their answers would be kept confidential, and that no one at their school or home would know how they responded. Honest responses to the questions were also encouraged. All procedures were approved by the University of Waterloo Office of Research Ethics and participating School Boards. More information on the COMPASS study methods and procedures can be found in print [33] or online [34].

### Measures

Moderate-to-vigorous physical activity (MVPA) was assessed with two questions including time per day spent doing moderate (e.g., walking, biking to school, recreational swimming) and hard (e.g., jogging, team sports, fast dancing, jump-rope) physical activities on each of the last 7 days. The scores for moderate and hard physical activities from each day were summed and divided by 7 to create an average minutes of MVPA/day score. Hours per day of MVPA was then calculated by dividing minutes/day by 60. Screen time was assessed with three questions including usual time per day spent watching/streaming TV shows or movies, playing video/computer games, and surfing the internet. The responses to the three questions were summed to create the screen time variable. Hours of screen time/day was then calculated by dividing minutes per day by 60. 1-week test-retest reliability intraclass correlation coefficients (ICC) for this scale have been reported as 0.75 for MVPA, 0.54 for watching TV shows/movies, 0.65 for video/computer games, and 0.71 for surfing the internet [35]. When compared to accelerometer-measured PA, the criterion validity ICCs were 0.22 for moderate PA, 0.18 for hard PA, and 0.25 for MVPA. These findings are comparable to other studies that examined the association between self-report PA measures and accelerometers [36].

Bullying was defined as physical attacks (e.g., getting beaten up, pushed, or kicked), verbal attacks (e.g., getting teased, threatened, or having rumors spread about you), cyber-attacks (e.g., being sent mean text messages or having rumors spread about you on the internet), and theft or damage of property. Frequency of peer victimization was assessed with one question: “In the last 30 days, how often have you been bullied by other students?” Response options included a) I have not been bullied by other students in the last 30 days, b) less than once a week, c) about once a week, d) 2 or 3 times a week, or e) daily or almost daily. For ease of interpretation peer victimization was collapsed into 2 categories including 1) was not bullied in the last 30 days and 2) was bullied in the last 30 days. These questions are similar to the “global” measure of peer victimization from the Olweus Bully/Victim Questionnaire [37] and to other adolescent population health surveys such as the Ontario Student Drug Use and Health Survey [38] and the Health Behavior in School-aged Children study which was conducted in 33 countries [16, 18].

Weight status was assessed using two self-reported height and weight questions [39] that are consistent with other large-scale surveys [40, 41]. Body mass index (BMI) was calculated as kg/m^2^ and age- and sex- specific non-overweight (coded as 0), overweight/obese weight status (coded as 1) categories were calculated based on World Health Organization standards [42]. In a validation study, the 1-week test-retest reliability ICCs were 0.96 for height, 0.99 for weight, and 0.95 for BMI [39]. Concurrent validity ICCs of self-reported and objectively measured values were 0.88 for height, 0.84 for weight, and 0.84 for BMI.

Covariates included grade, ethnicity/race, weekly spending money, and future education plans. Ethnicity/race was assessed with the question “How would you describe yourself?” (mark all that apply). Responses were collapsed into White, Black, Asian, Aboriginal (First Nations, Metis, Inuit), Latin American/Hispanic, and mixed/other.

Because adolescents are not necessarily aware of their household income and the education levels of their parents [43], weekly spending money and future education plans were used as indicators of personal economic status. Adolescents whose parents have attained a higher education tend to have a higher disposable income in terms of weekly allowance and job income [44], and weekly spending money has been shown to be positively associated with vigorous exercise and watching TV among adolescents [43]. Research in Norway found that plans for higher education were highly stable across adolescence, and the participants’ educational plans tended to correspond well with their parents education [45]. Weekly spending money was assessed with the question “About how much money do you usually get each week to spend on yourself or to save?”, and included money from allowances and jobs like babysitting and delivering papers. To be consistent with other COMPASS studies, [46–49] and in order to retain as many cases as possible, this variable was collapsed into “zero”, “$1–20”, “$21–100”, “> $100”, and “don’t know”. Future education plans was assessed with the question “What is the highest level of education you think you will get?” with six response options including completed high school or less; college/trade/vocational certificate; university bachelor’s degree; university master’s/PhD/law school/medical school/teachers’ college degree; and I don’t know.

### Analysis

Preliminary analyses were completed using IBM SPSS Version 22. Univariate outliers for the dependent variables with a *z*-score above 3 or below −3 (screen time = 463 cases, MVPA = 335 cases) were coded as missing. A further 481 multivariate outliers (all standardized residual >3) for screen time and 191 multivariate outliers (all standardized residuals >3) for MVPA were detected and coded as missing. Coding the outliers as missing allowed these values to be estimated in the main analysis. The assumptions of homoscedasticity and multivariate normality were met. The error variance also appeared to be similar across schools. An inspection of the bivariate correlations showed no evidence of multicollinearity (i.e., *r*’s < .70 and VIF < 10).

Multilevel path analysis, controlling for clustering by schools, was used to test the multiple meditation model. Mediation was examined using the product of coefficient method (Cerin & MacKinnon, 2009). It involved estimating 1) the associations between weight status and peer victimization (α path coefficient), 2) the association between peer victimization and the outcome variables while controlling for weight status (β path coefficient), and 3) the mediated effect (αβ path coefficient). Though previous methods required a significant pathway between the predictor and outcome variables to proceed with mediation analysis, new procedures do not require this step [50]. However, both the total effects (i.e., association between the predictor and outcome variables) and direct effects (i.e., the association between the predictor and outcome variables with the indirect effect removed) will still be presented. The mediated effect (or indirect effect) is the estimated effect of weight status on MVPA and weight status on screen time through peer victimization. Because weight status is dichotomous, the indirect effect can be interpreted as the mean difference between groups (non-overweight vs overweight/obese) in units of the outcome (MVPA, screen time) attributable to the pathway through peer victimization [51]. The significance of the mediation effect *p* < .05 and the 95% confidence intervals provided evidence of mediation [50].

The path analysis was computed in Mplus Version 7.1 using the WLSMV estimator, which employs “weighted least square parameter estimates using a diagonal matrix with standard errors and a mean- and variance-adjusted chi-square test statistic that use a full weight matrix” [52]. Probit regression was used to test associations between the control variables and peer victimization, and body weight status and peer victimization. Linear regression was used to test all associations with screen time and MVPA. This resulted in a fully saturated model and therefore model fit statistics were not available. Grade, ethnicity/race, weekly spending money, and future education plans were added as control variables by including them as exogenous variables predicting all outcome variables in the model. In a preliminary analysis, differences by gender were explored within the proposed model. When comparing differences by subgroups, formal tests of moderation are recommended [53]. If significant differences exist, stratification by groups is justified. Thus, to test for gender moderation on each pathway, interaction terms were created between weight status and gender, and weight status and peer victimization. The interaction terms were then tested for their effect on the outcome variables one by one within the model, with gender included as a main effect. Significant differences were found on two pathways; therefore, the model is presented separately for males and females.

All of the outcome variables were missing on less than 5% of the cases, and 2.5% of the total cases in the dataset were missing. Missingness on the outcome variables was predicted by multiple variables including variables from the larger dataset that are not part of the main analysis. We therefore assumed that the data was missing at random and estimated the missing cases using full-information maximum likelihood. The variables predictive of missingness but not included in the analysis (i.e., participation in school and non-school sports, whether the last week was a typical week for PA, perceived support for bullying from the school) were added as auxiliary variables. Cases missing on all variables (*n* = 13) or one of the x-variables (i.e., weight status and all covariates; *n* = 6142) were excluded from the analysis. This resulted in a final sample size of 18,147 participants.

## Results

Table 1 presents the sociodemographic information. Approximately half of the sample was female (49%) and 73-75% were white. Table 2 presents the descriptive information for the model variables. Specifically, 19% of females and 32% of males were overweight or obese and 21% of females and 15% of males had been victimized at least once during the last 30 days. On average, females reported 4.5 h per day of screen time and 1.8 h per day of MVPA and males reported 5.2 h per day of screen time and 2.2 h per day of MVPA.

**Table 1:** Sociodemographic information

Characteristic	Females (*n* = 8904)	Males (*n* = 9243)
Grade – count (%)		
9	2112 (23.7)	2233 (24.2)
10	2336 (26.2)	2305 (24.9)
11	2255 (25.3)	2333 (25.2)
12	2201 (24.7)	2372 (25.7)
Ethnicity/race – count (%)		
White	6653 (74.7)	6701 (72.5)*
Black	269 (3.0)	420 (4.5)*
Asian	522 (5.9)	503 (5.4)
Aboriginal	210 (2.4)	257 (2.8)
Latino/Hispanic	164 (1.8)	223 (2.4)*
Other/Mixed	1086 (12.2)	1139 (12.3)
Anticipated education level – count (%)		
High school diploma or graduation equivalency or less	328 (3.7)	471 (5.1)*
College/trade/vocational certificate	1754 (19.7)	2874 (31.1)*
University Bachelor’s degree	2317 (26.0)	2289 (24.8)
Master’s/PhD/law school/medical degree/teachers’ college degree	3212 (36.1)	2410 (26.1)*
I don’t know	1293 (14.5)	1199 (13.0)*
Weekly spending money – count (%)		
Zero	1229 (13.8)	1443 (15.6)*
$1–20	2688 (30.2)	2758 (29.8)
$21–100	2743 (30.8)	2431 (26.3)*
$100+	1205 (13.5)	1572 (17.0)*
I don’t know	1039 (11.7)	1039 (11.2)

Differences by gender tested via chi-square tests of independence

*indicates significant differences (*p* < .05) between males and females

**Table 2:** Descriptive statistics for the main model variables

Characteristic	Females (*n* = 8904)	Males (*n* = 9243)
Weight status – count (%)		
Non-Overweight	7189 (80.7)	6312 (68.3)*
Overweight/Obese	1715 (19.3)	2931 (31.7)
Peer victimization – count (%)		
None	7012 (79.2)	7781 (85.0)*
At least once in the past 30 days	1841 (20.8)	1371 (15.0)
Daily screen time – mean hours/day (SD)	4.500 (2.675)	5.231 (2.729)*
Daily MVPA – mean hours/day (SD)	1.758 (1.146)	2.175 (1.262)*

*MVPA* moderate-to-vigorous physical activity

Differences by gender tested via chi-square tests of independence (weight status, peer victimization), and independent samples *t*-tests (screen time, MVPA)

Numbers in the table may not tally to the total *N* due to missing data

*indicates significant differences (*p* < .05) between females and males

### Gender differences

Significant gender differences were found for two pathways. Specifically, gender moderated the association between peer victimization and screen time (B = 0.380 ± 0.073, *p* < .001), with females having a stronger association than males. The association between weight status and screen time was also moderated by gender (B = −0.368 ± 0.098, *p* < .001), with females having a stronger association than males.

### Path analysis - females

**Fig. 1:**
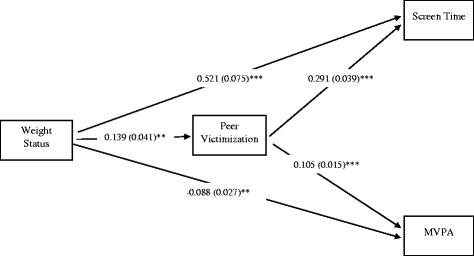
The final model for females with unstandardized beta values and standard errors. Non-significant pathways are indicated by a dotted line. Weight status is coded as “non-overweight” = 0, “overweight/obese” = 1. Peer victimization is coded as “has not been bullied in the last 30 days” = 0, “has been bullied at least once in the last 30 days” = 1. MVPA = moderate-to-vigorous physical activity. *p* < .05; **p < .01; ***p < .001. Model was adjusted for grade, ethnicity/race, weekly spending money, and future education plans

The full model for females including unstandardized coefficients and standard errors is presented in Fig. 1. All analyses adjusted for grade, ethnicity/race, weekly spending money, and future education plans. Weight status was positively associated with peer victimization (α path coefficient; B = 0.139 ± 0.041, *p* = 0.001). Peer victimization was positively associated to screen time (β coefficient; B = 0.291 ± 0.039, *p* < .001). Further, 8% (R^2^ = .083) of the variance was explained for screen time; however, was reduced to 2% (R^2^ = .024) when the covariates were removed.

The total, direct and indirect effects are presented in Table 3. The total effect of weight status on screen time was significant and indicates that the females with overweight/obesity participated in 0.562 more hours per day (or 34 min per day) of screen time than the females who were not overweight (± 0.074, *p* < .001). When the indirect effects were taken into account, the direct pathway from weight status to screen time remained significant (B = 0.521 ± 0.075, *p* < .001). The indirect effect of weight status on screen time through peer victimization was significant. Specifically, there was 0.040 additional hours per day (or 2 min per day) of screen time in the females with overweight/obesity compared to females who were not overweight that could be attributed to increased peer victimization (± 0.014, *p* = .004). Therefore, our first hypothesis that peer victimization mediates the positive association between weight status and screen time was partially supported. Greater peer victimization may partially explain why adolescents with overweight and obesity engage in more minutes of screen time than those who are not overweight. However, the effects are very small and the practical significance of such findings are questionable.

**Table 3:** Unstandardized path coefficients for direct, total indirect, specific indirect, and total effects (*N* = 18,147)

	Model Outcomes					
	Screen Time (hours/day)			MVPA (hours/day)		
	Coefficient (SE)	*p*-value	95% CI	Coefficient (SE)	*p*-value	95% CI
Females						
Direct Effects	0.521 (0.075)	<.001	0.375, 0.668	−0.088 (0.027)	.001	−0.141, −0.036
Indirect Effects	0.040 (0.014)	.004	0.013, 0.068	0.015 (0.004)	.001	0.006, 0.023
Total Effects	0.562 (0.074)	<.001	0.419, 0.708	−0.074 (0.027)	.006	−0.127, −0.021
Males						
Direct Effects	0.194 (0.053)	<.001	0.090, 0.298	−0.058 (0.027)	.034	−0.112, −0.004
Indirect Effects	0.019 (0.008)	.012	0.004, 0.035	0.003 (0.002)	.197	−0.001, 0.007
Total Effects	0.214 (0.053)	<.001	0.109, 0.319	−0.056 (0.027)	.043	−0.109, −0.002

Unstandardized path coefficients are presented

*MVPA* moderate-to-vigorous physical activity

Weight status is coded as 0 = non-overweight, 1 = overweight/obese

Model was adjusted for grade, ethnicity/race, weekly spending money, and future education plans

As mentioned previously, weight status was positively associated with peer victimization (α path coefficient; B = 0.139 ± 0.041, *p* = 0.001). Unexpectedly, peer victimization was *positively* associated with MVPA (β coefficient; B = 0.105 ± 0.015, *p* < .001). Further, 5% (R^2^ = .049) of the variance was explained for MVPA; however, these proportions were reduced to 1% (R^2^ = .013) for MVPA when the covariates were removed. The pathway between weight status and MVPA (i.e., total effect) was significant for females. This indicates that females with overweight/obesity participated in 0.074 less hours per day (or 4 min per day) of MVPA than the females who were not overweight (± 0.027, *p* = .006). When the indirect effects were accounted for, the direct effect between MVPA and weight status remained significant (*p* = .001). The indirect effect through peer victimization was also significant. Specifically, there was 0.015 additional hours per day (or 1 min per day) of MVPA in the females who were overweight/obese compared to the females that were not overweight that could be attributed to increased peer victimization (± 0.004, *p* = .001). Therefore, our second hypothesis was not supported for females. Females with overweight and obesity did engage in less MVPA and were more likely to have been victimized compared to the adolescents who were not overweight; however, those who were victimization tended to perform more MVPA.

### Path analysis – Males

**Fig. 2:**
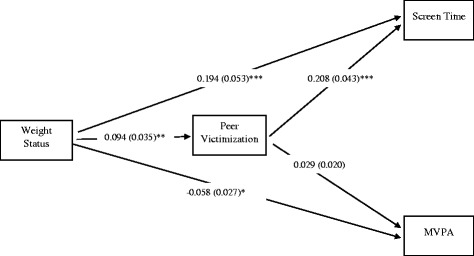
The final model for males with unstandardized beta values and standard errors. Non-significant pathways are indicated by a dotted line. Weight status is coded as “non-overweight” = 0, “overweight/obese” = 1. Peer victimization is coded as “has not been bullied in the last 30 days” = 0, “has been bullied at least once in the last 30 days” = 1. MVPA = moderate-to-vigorous physical activity. *p < .05; ** *p* < .01;***p < .001. Model was adjusted for grade, ethnicity/race, weekly spending money, and future education plans

The full model for males including unstandardized coefficients and standard errors is presented in Fig. 2. All analyses adjusted for grade, ethnicity/race, weekly spending money, and future education plans. Weight status was positively associated with peer victimization (α path coefficient; B = 0.094 ± 0.035, *p* = 0.008). Controlling for weight status, peer victimization was positively associated with screen time (β coefficients; B = 0.208 ± 0.043, *p* < .001). Further, 4% (R^2^ = .041) of the variance was explained for screen time, however these proportions were reduced to 0.9% (R^2^ = .009) for screen time when the covariates were removed.

The total, direct and indirect effects for the male model are presented in Table 3. The pathway between weight status and screen time (i.e., total effect) was significant for males. This indicates that the males with overweight/obesity participated in 0.214 more hours per day (or 13 min per day) of screen time than the males who were not overweight (± 0.053, *p* < .001). When the indirect effects were accounted for, the direct effect from weight status and screen time remained significant (*p* < .001). The indirect effect through peer victimization was also significant (B = 0.019 ± 0.008, *p* = .012). Specifically, there was 0.019 additional hours per day (or 1 min per day) of screen time in the males with overweight/obesity compared to the males who were not overweight that could be attributed to increased peer victimization. Thus, our first hypothesis that peer victimization mediates the positive association between weight status and screen time was partially supported in males. Greater peer victimization may partially explain why males who are overweight or obese engage in more minutes of screen time than do males who are not overweight. However, the effects are very small and thus may not have practical significance.

As mentioned, weight status was positively associated with peer victimization (α path coefficient; B = 0.094 ± 0.035, *p* = 0.008). Controlling for weight status, peer victimization was not associated with MVPA (B = 0.029 ± 0.020, *p* = 0.149). Further, the model explained 4% (R^2^ = .035) of the variance in MVPA; however, these proportions were reduced to 0.1% (R^2^ = .001) for MVPA when the covariates were removed. The pathway between weight status and MVPA (i.e., total effect) was significant for males. This indicates that males with overweight/obesity participated in 0.056 less hours per day of MVPA (or 3 min per day) than males who were not overweight (± 0.027, *p* = .043). When the indirect effects were accounted for, the association between weight status and MVPA (i.e., direct effect) remained significant (*p* = .034). The indirect effect through peer victimization was not significant (*p* = .197). Thus, our second hypothesis that peer victimization mediates the positive association between weight status and MVPA was not supported.

## Discussion

Our study is among the first to empirically examine whether negative peer experiences help explain why adolescents who are overweight and obese tend to engage in more screen time and be less active than those who are not overweight. Previous research has shown that overweight and obese adolescents tend to have fewer friendship nominations and to be on the periphery of friendship networks [54]. These individuals are often victims of bullying [16–19], and this type of conflict is known to have negative effects on psychological health [55]. Consistent with the model proposed by Salvy et al. [12], our findings suggest that in both males and females the association between weight status and higher levels of screen time is partially explained by peer victimization. Specifically, we found evidence of partial mediation whereby females with overweight/obesity reported 34 more minutes of screen time per day than did females who were not overweight and 2 of these minutes could be attributed to experiencing peer victimization. Similarly, males who were overweight/obese reported 13 more minutes of screen time per day than the males who were not overweight and 1 of these minutes could be attributed to experiencing more victimization. Males and females who were overweight/obese also reported less MVPA compared to those who were not overweight; however, peer victimization did not mediate these associations in the hypothesized direction.

Similarly, Vanderwater et al. [56] found that adolescents with overweight/obesity spent less time with friends, which resulted in them being less active, and subsequently led to spending more time watching TV. This study along with ours highlights some of the peer difficulties (i.e., peer victimization, less time spent with friends) adolescents with overweight/obesity face and how these issues can influence their screen-related activities. It is also consistent with a growing body of literature suggesting that weight-related stigma and peer difficulties experienced by individuals who are overweight negatively impact health behaviors [12].

Though the overall findings were similar for males and females, a few differences were observed. Specifically, stronger associations were observed between weight status and screen time, and peer victimization and screen time for females (yet still significant for males). Higher rates of peer victimization were also observed for females (21%) compared to males (15%). It is possible the psychological impacts of peer victimization are slightly more harmful to females compared to males, or that the type of victimization experienced by females tends to be more detrimental. For example, across studies females who are overweight/obese have a lower quality of life than do males who are overweight/obese [57]. Further, females are more often teased about their weight, and report being more bothered by these experiences compared to males [58]. Females may also feel more pressure to conform to societal body ideals, and thus peer victimization could have greater impacts on body image and consequently their health and health behaviors [59]. Dating may be one source of this pressure as obese females are less likely to date than their peers, yet this difference is not seen in males [17]. Further, females tend to report greater bullying when they believe their body is too fat; whereas, boys report greater bullying when their believe their body is too thin [16].

We do acknowledge that the mediation effect of peer victimization on the weight status-screen time association is small. Some studies have found that adolescents with overweight and obesity underreport their weight compared to healthy weight adolescents, which can result in a misclassification of adolescents with overweight as healthy weight [60]. Therefore, it is possible that some of the overweight participants were misclassified as non-overweight thereby reducing the magnitude of the associations. Indeed, we found the rates of overweight/obesity in this sample (25.6%) to be lower than national rates in Canada (33.2%) [61]. However, the format of the COMPASS height and weight questions are slightly different than previous surveys, which may influence findings. For example, in the review by Sherry et al. [60] females were found to underreport their weight more than males, yet this bias was not found in the COMPASS survey [39]. Also, a recent meta-analysis found that the strength of association between weight status and peer victimization did not differ between studies that used self-report vs. objective measures of height and weight, suggesting that self-reported weight status does not bias this association [21].

Further, it should be mentioned that when peer victimization was taken into account, there was still a direct association between weight status and screen time in both males and females. This suggests that there are other unmeasured mechanism(s) that explain these associations. As previously mentioned, Vanderwater and co-authors [56] found that time spent with friends was an important mediator of the weight status-TV time association; therefore, future research will benefit from examining the individual and combined impacts of friendship (e.g., presence of a friend, number of friends, time spent with friends) and negative peer experiences. Other research has found that having a best friend buffers a child from the negative psychosocial consequences of peer victimization [62]; therefore, another possible avenue to explore is whether having one or more friends negates or reduces the negative impacts of peer victimization on screen time.

The findings of this study were not consistent between screen time and MVPA. Although weight status was negatively associated with MVPA as hypothesized, peer victimization was positively associated with MVPA in females, and unassociated in males. Consequently, it did not mediate the weight status-MVPA association in the hypothesized direction. This is surprising considering peer victimization is associated with lower PA and PE attendance [25–27], and adolescents with overweight/obesity describe victimization as a barrier to PA participation [13]. Again, the potential underreporting of weight (and subsequently BMI) [60] in the participants with overweight and obesity could have attenuated the findings. Another potential explanation is that participants with overweight/obesity overreported their PA compared to the participants who were not overweight, however this phenomenon is less consistent in the literature [63, 64]. Finally, it is possible that some adolescents with overweight/obesity may be more resilient and less affected by victimization from their peers [65]. For instance, Faith and colleagues [28] found that children who were criticized for their weight participated in less mild-intensity PA but this association was moderated by problem-focused coping skills, such that weight criticism did not lead to lower PA in children who could cope with the criticisms. As this is one of the first studies to test the model proposed by Salvy et al. [11], and because the associations between weight status, peer victimization, and MVPA are supported by previous research and theory, we suggest that researchers continue to examine these associations in greater detail among both children and adolescents.

Future research will be important for advancing the theory around weight status, negative peer experiences, and PA, sedentary behavior, and screen time. Studies should investigate whether the impact of peer victimization on PA and screen time is specific to weight-based teasing (rather than general peer victimization), and examine the full range of negative peer experiences that young people encounter (e.g., peer rejection, lack of friends, ostracism, loneliness). Ecological momentary assessment and natural observations in PE classes and playgrounds could be used to investigate whether PA tends to decrease, and/or sedentary behavior increases immediately after a peer victimization experience. Further, studies should examine these associations using an objective measure of sedentary behavior (e.g., inclinometer, accelerometer) in addition to a screen time measure, and ideally adopt longitudinal designs. When the data collection is complete, the COMPASS study will have four waves of data, and we will be able to examine these associations across multiple time points.

The strengths of our study include the large sample and the wide age range of participants (grades 9–12). The multilevel multiple path analysis allowed us to control for clustering within schools, and to examine multiple outcome variables simultaneously. Additionally, some limitations should also be noted. First, the study is cross-sectional, and thus we cannot be certain that the paths in the model are specified accurately. In the absence of temporal precedence, it is recommended that the causal sequence be informed by theory [66]. Future research using additional waves of the COMPASS study will allow us to establish the temporal sequence of the associations. Second, all of the measures were self-reported, which are known to have associated biases, and this could have impacted study findings. Further, though we recognize that a multiple item measure of peer victimization would have been ideal, the one-item measure used in this study is consistent with most population-based studies in the health literature [18, 67]. In addition, the use of self-report measures allowed the COMPASS study to collect information from a very large sample of students, where objective measures are not feasible within the passive consent protocol required for collecting substance use data. Also, our model only examined peer victimization, and did not assess the full range of potential negative peer experiences that young people encounter. Similarly, we only assessed screen time and thus the findings cannot be generalized to all sitting-related behaviors or sedentary behavior. Finally, despite the large sample size, this was a convenient sample of schools in Ontario and thus the findings may not generalize to all schools and students in Ontario.

## Conclusion

Peer victimization partially explains why adolescents with overweight and obesity engage in higher levels of screen time than adolescents who are not overweight. This is one of the first studies to investigate the impacts of peer victimization on the health behaviors of adolescents, and thus more research is needed before bully prevention or conflict resolution training are explored as intervention strategies. The use of objective measures and longitudinal designs, and examining the immediate impact of peer victimization within specific contexts will be useful for progressing this topic area.

## Abbreviations

BMI: Body mass index
MVPA: Moderate-to-vigorous physical activity
PA: Physical activity

## References

[1] Poitras VJ, Gray CE, Borghese MM, Carson V, Chaput JP, Janssen I (2016). Systematic review of the relationships between objectively measured physical activity and health indicators in school-aged children and youth. Appl Physiol Nutr Metab..

[2] Janssen I, LeBlanc AG (2010). Systematic review of the health benefits of physical activity and fitness in school-aged children and youth. IJBNPA..

[3] Tremblay MS, Carson V, Chaput J-P, Connor Gorber S, Dinh T, Duggan M (2016). Canadian 24-hour movement guidelines for children and youth: an integration of physical activity, sedentary behaviour, and sleep. Appl Physiol Nutr Metab.

[4] Carson V, Hunter S, Kuzik N, Gray CE, Poitras V J, Chaput J-P, et al. Systematic review of sedentary behaviour and health indicators in school-aged children and youth: an update 1. Appl Physiol, Nutr, and Metab. 2016;41:S240-S265. 10.1139/apnm-2015-0630 27306432

[5] ParticipACTION. PartcipACTION report on physical activity of children and youth. 2016. http://stage.participaction.com/sites/default/files/downloads/2016%20ParticipACTION%20Report%20Card%20-%20Full%20Report.pdf. Accessed 7 June 2016.

[6] Janssen I, Katzmarzyk PT, Boyce WF, Vereecken C, Mulvihill C, Roberts C (2005). Comparison of overweight and obesity prevalence in school-aged youth from 34 countries and their relationships with physical activity and dietary patterns. Obes Rev.

[7] Janssen I, Katzmarzyk PT, Boyce WF, King MA, Pickett W (2004). Overweight and obesity in Canadian adolescents and their associations with dietary habits and physical activity patterns. J of Adolesc Health.

[8] Biddle SJ, Pearson N, Ross GM, Braithwaite R (2010). Tracking of sedentary behaviours of young people: a systematic review. Prev Med.

[9] Craigie AM, Lake AA, Kelly SA, Adamson AJ, Mathers JC (2011). Tracking of obesity-related behaviours from childhood to adulthood: a systematic review. Maturitas.

[10] Telama R (2009). Tracking of physical activity from childhood to adulthood: a review. Obes Facts.

[11] Salvy S-J, Bowker JC, Germeroth L, Barkley J (2012). Influence of peers and friends on overweight/obese youths’ physical activity. Exerc Sport Sci Rev.

[12] Puhl R, Suh Y (2015). Health consequences of weight stigma: implications for obesity prevention and treatment. Curr Obes Rep.

[13] Stankov I, Olds T, Cargo M (2012). Overweight and obese adolescents: what turns them off physical activity?. IJBNPA..

[14] Volk AA, Dane AV, Marini ZA (2014). What is bullying? A theoretical redefinition. Dev Rev.

[15] Kowalski RM, Limber SP (2013). Psychological, physical, and academic correlates of cyberbullying and traditional bullying. J Adolesc Health.

[16] Brixval CS, Rayce SL, Rasmussen M, Holstein BE, Due P (2011). Overweight, body image and bullying—an epidemiological study of 11-to 15-years olds. Eur J Pub Health.

[17] Pearce MJ, Boergers J, Prinstein MJ (2002). Adolescent obesity, overt and relational peer victimization, and romantic relationships. Obes Res.

[18] Janssen I, Craig WM, Boyce WF, Pickett W (2004). Associations between overweight and obesity with bullying behaviors in school-aged children. Pediatrics.

[19] Lumeng JC, Forrest P, Appugliese DP, Kaciroti N, Corwyn RF, Bradley RH (2010). Weight status as a predictor of being bullied in third through sixth grades. Pediatrics.

[20] Ottova V, Erhart M, Rajmil L, Dettenborn-Betz L, Ravens-Sieberer U (2012). Overweight and its impact on the health-related quality of life in children and adolescents: results from the European KIDSCREEN survey. Qual Life Res.

[21] Van Geel M, Vedder P, Tanilon J (2014). Are overweight and obese youths more often bullied by their peers? A meta-analysis on the relation between weight status and bullying. Int J Obes.

[22] Juvonen J, Graham S (2014). Bullying in schools: the power of bullies and the plight of victims. Annu Rev Psychol.

[23] Puhl RM, Luedicke J, Heuer C (2011). Weight-based victimization toward overweight adolescents: observations and reactions of peers. J Sch Health..

[24] Puhl RM, Peterson JL, Luedicke J (2013). Weight-based victimization: bullying experiences of weight loss treatment–seeking youth. Pediatrics.

[25] Henriksen P, Rayce S, Melkevik O, Due P, Holstein B. Social background, bullying, and physical inactivity: national study of 11-to 15-year-olds. Scand J Med Sci Spor. 2015; doi:10.1111/sms.12574. 10.1111/sms.12574 26454139

[26] Roman CG, Taylor CJ (2013). A multilevel assessment of school climate, bullying victimization, and physical activity. J Sch Health..

[27] Storch EA, Milsom VA, DeBraganza N, Lewin AB, Geffken GR, Silverstein JH (2007). Peer victimization, psychosocial adjustment, and physical activity in overweight and at-risk-for-overweight youth. J Pediatr Psychol.

[28] Faith MS, Leone MA, Ayers TS, Heo M, Pietrobelli A (2002). Weight criticism during physical activity, coping skills, and reported physical activity in children. Pediatrics.

[29] Hertz MF, Everett Jones S, Barrios L, David-Ferdon C, Holt M (2015). Association between bullying victimization and health risk behaviors among high school students in the United States. J Sch Health.

[30] Demissie Z, Lowry R, Eaton DK, Hertz MF, Lee SM. Associations of school violence with physical activity among US high school students. J Phys Act Health. 2014;11 10.1123/jpah.2012-0191 PMC10947244 25078515

[31] Rose BM, Holmbeck GN, Coakley RM, Franks EA (2004). Mediator and moderator effects in developmental and behavioral pediatric research. J Dev Behav Pediatr.

[32] Dishman RK, Hales DP, Pfeiffer KA (2006). Physical self-concept and self-esteem mediate cross-sectional relations of physical activity and sport participation with depression symptoms among adolescent girls. Health Psychol.

[33] Leatherdale ST, Brown KS, Carson V (2014). The COMPASS study: a longitudinal hierarchical research platform for evaluating natural experiments related to changes in school-level programs, policies and built environment resources. BMC Public Health.

[34] COMPASS System. https://uwaterloo.ca/compass-system/compass-system-projects/compass-study. Accessed 7 July 2016.

[35] Leatherdale ST, Laxer RE, Faulkner G (2014). Reliability and validity of the physical activity and sedentary behaviour measures in the COMPASS questionnaire.

[36] Chinapaw MJM, Mokkink LB, van Poppel MNM, van Mechelen W, Terwee CB (2010). Physical activity questionnaires for youth. Sports Med.

[37] Solberg ME, Olweus D (2003). Prevalence estimation of school bullying with the Olweus bully/victim questionnaire. Aggress Behav.

[38] Sampasa-Kanyinga H, Willmore J (2015). Relationships between bullying victimization psychological distress and breakfast skipping among boys and girls. Appetite.

[39] Leatherdale ST, Laxer RE (2013). Reliability and validity of the weight status and dietary intake measures in the COMPASS questionnaire: are the self-reported measures of body mass index (BMI) and Canada’s food guide servings robust. IJBNPA.

[40] Brener ND, Mcmanus T, Galuska DA, Lowry R, Wechsler H (2003). Reliability and validity of self-reported height and weight among high school students. J Adolesc Health.

[41] Wong SL, Leatherdale ST, Manske SR (2006). Reliability and validity of a school-based physical activity questionnaire. Med Sci Sports Exerc.

[42] Onis Md, Onyango AW, Borghi E, Siyam A, Nishida C, Siekmann J. Development of a WHO growth reference for school-aged children and adolescents. Bull World Health Organization. 2007;85:660–7. 10.2471/BLT.07.043497 PMC2636412 18026621

[43] Currie CE, Elton RA, Todd J, Platt S (1997). Indicators of socioeconomic status for adolescents: the WHO health behaviour in school-aged children survey. Health Educ Res.

[44] Soteriades ES, DiFranza JR (2003). Parent's socioeconomic status, adolescents' disposable income, and adolescents' smoking status in Massachusetts. Am J Public Health.

[45] Friestad C, Lien N, Klepp K-I (2001). Educational plans-when are they establis hed? Implications for the measurement of socio-economic status in youth. Young.

[46] Herciu AC, Laxer RE, Cole A, Leatherdale ST. A cross-sectional study examining factors associated with youth binge drinking in the COMPASS study: year 1 data. J Alcohol Drug Depend. 2014;172

[47] Reid JL, Hammond D, McCrory C, Dubin JA, Leatherdale ST (2015). Use of caffeinated energy drinks among secondary school students in Ontario: prevalence and correlates of using energy drinks and mixing with alcohol. Can J Public Health.

[48] Leatherdale ST (2015). An examination of the co-occurrence of modifiable risk factors associated with chronic disease among youth in the COMPASS study. Cancer Causes Control.

[49] Leatherdale ST, Harvey A (2015). Examining communication-and media-based recreational sedentary behaviors among Canadian youth: results from the COMPASS study. Prev Med.

[50] Cerin E, MacKinnon DP (2009). A commentary on current practice in mediating variable analyses in behavioural nutrition and physical activity. Public Health Nutr.

[51] Hayes AF (2009). Beyond baron and Kenny: statistical mediation analysis in the new millennium. Commun Monogr.

[52] Muthen LK, Muthen BO (1998). Mplus user’s guide.

[53] Atkin AJ, van Sluijs EMF, Dollman J, Taylor WC, Stanley RM (2016). Identifying correlates and determinants of physical activity in youth: how can we advance the field?. Prev Med.

[54] Strauss RS, Pollack HA (2003). Social marginalization of overweight children. Arch Pediatr Adolesc Med..

[55] Reijntjes A, Kamphuis JH, Prinzie P, Telch MJ (2010). Peer victimization and internalizing problems in children: a meta-analysis of longitudinal studies. Child Abuse Negl.

[56] Vandewater EA, Park SE, Hébert ET, Cummings HM. Time with friends and physical activity as mechanisms linking obesity and television viewing among youth. IJBNPA. 2015;12 Suppl 1:S6. 10.1186/1479-5868-12-S1-S6 PMC4519112 26221737

[57] Buttitta M (2014). Lliescu C, Rousseau a, Guerrien a. Quality of life in overweight and obese children and adolescents: a literature review. Qual Life Res.

[58] Neumark-Sztainer D, Falkner N, Storey M, Perry C (2002). Hannan, PJ. Mulert S Weight-teasing among adolescents: correlations with weight status and disordered eating behaviors Int J Obes.

[59] Voelker DK, Reel JJ, Greenleaf C (2015). Weight status and body image perceptions in adolescents: current perspectives. Adoles Health Med Ther.

[60] Sherry B, Jefferds ME, Grummer-Strawn LM (2007). Accuracy of adolescent self-report of height and weight in assessing overweight status: a literature review. Arch Pediatr Adolesc Med.

[61] Shields M, Tremblay MS (2010). Canadian childhood obesity estimates based on WHO, IOTF and CDC cut-points. IJPO.

[62] Hodges EVE, Boivin M, Vitaro F, Bukowski WM (1999). The power of friendship: protection against an escalating cycle of peer victimization. Dev Psychol.

[63] McMurray RG, Ward DS, Elder JP, Lytle LA, Strikmiller PK, Bagget CD (2008). Do overweight girls overreport physical activity?. Am J Health Behav.

[64] Slootmaker SM, Schuit AJ, Chinapaw MJ, Seidell JC, Van Mechelen W (2009). Disagreement in physical activity assessed by accelerometer and self-report in subgroups of age, gender, education and weight status. IJBNPA..

[65] Russell-Mayhew S, McVey G, Bardick A, Ireland A. Mental health, wellness, and childhood overweight/obesity. J Obes. 2012; doi:10.1155/2012/281801. 10.1155/2012/281801 PMC3388583 22778915

[66] MacKinnon DP (2008). Introduction to statistical mediation analysis. Chapter 3.

[67] Craig W, Harel-Fisch Y, Fogel-Grinvald H, Dostaler S, Hetland J, Simons-Morton B (2009). A cross-national profile of bullying and victimization among adolescents in 40 countries. Int J Public Health.

